# Prevalence and determinants of diabetes and prediabetes in southwestern Iran: the Khuzestan comprehensive health study (KCHS)

**DOI:** 10.1186/s12902-021-00790-x

**Published:** 2021-06-29

**Authors:** Sanam Hariri, Zahra Rahimi, Nahid Hashemi-Madani, Seyyed Ali Mard, Farnaz Hashemi, Zahra Mohammadi, Leila Danehchin, Farhad Abolnezhadian, Aliasghar Valipour, Yousef Paridar, Mohammad Mahdi Mir-Nasseri, Alireza Khajavi, Sahar Masoudi, Saba Alvand, Bahman Cheraghian, Ali Akbar Shayesteh, Mohammad E. Khamseh, Hossein Poustchi

**Affiliations:** 1 Liver and Pancreatobiliary Diseases Research Center, Digestive Diseases Research Institute, Shariati Hospital, Tehran University of Medical Sciences, N. Kargar St, Tehran, Iran; 2 Hearing Research Center, Department of Biostatistics and Epidemiology, School of Public Health, Ahvaz Jundishapur University of Medical Sciences, Ahvaz, Iran; 3 Endocrine Research Center, Institute of Endocrinology and Metabolism, Iran University of Medical Sciences, Tehran, Iran; 4 Alimentary Tract Research Center, Imam Khomeini Hospital Clinical Research Development Unit, School of Medicine, Ahvaz Jundishapur University of Medical Sciences, Ahvaz, Iran; 5 Faculty of Medicine, Behbahan University of Medical Sciences, Behbahan, Iran; 6 Ahvaz Jundishapur University of Medical Sciences, Ahvaz, Iran; 7 Shoushtar School of Medical Sciences, Shoushtar, Iran; 8 Abadan Faculty of Medical Sciences, Abadan, Iran; 9 Faculty of Medicine, Dezful University of Medical Sciences, Dezful, Iran; 10 Digestive Diseases Research Institute, Tehran University of Medical Sciences, Tehran, Iran; 11 Student Research Committee, Faculty of Paramedical Sciences, Shahid Beheshti University of Medical Sciences, Tehran, Iran; 12 Alimentary Tract Research Center, Imam Khomeini Hospital Clinical Research Development Unit, Department of Biostatistics and Epidemiology, School of Public Health, Ahvaz Jundishapur University of Medical Sciences, Ahvaz, Iran; 13 Alimentary Tract Research Center, Clinical Sciences Research Institute, Ahvaz Jundishapur University of Medical Sciences, Ahvaz, Iran

**Keywords:** Diabetes, Prediabetes, Epidemiology, Risk-factors, Low and middle-income countries, Middle-east, Iran

## Abstract

**Background:**

The Middle East and North Africa (MENA) is postulated to have the highest increase in the prevalence of diabetes by 2030; however, studies on the epidemiology of diabetes are rather limited across the region, including in Iran.

**Methods:**

This study was conducted between 2016 and 2018 among Iranian adults aged 20 to 65 years residing in Khuzestan province, southwestern Iran. Diabetes was defined as the fasting blood glucose (FBG) level of 126 mg/dl or higher, and/or taking antidiabetic medications, and/or self-declared diabetes. Prediabetes was defined as FBG 100 to 125 mg/dl. Multinomial logistic regression models were used to examine the association of multiple risk factors that attained significance on the outcome.

**Results:**

Overall, 30,498 participants were recruited; the mean (±SD) age was 41.6 (±11.9) years. The prevalence of prediabetes and diabetes were 30.8 and 15.3%, respectively. We found a similar prevalence of diabetes in both sexes, although it was higher among illiterates, urban residents, married people, and smokers. Participants aged 50–65 and those with Body Mass Index (BMI) 30 kg/m^2^ or higher were more likely to be affected by diabetes [RR: 20.5 (18.1,23.3) and 3.2 (3.0,3.6)]. Hypertension [RR: 5.1 (4.7,5.5)], waist circumference (WC) equal or more than 90 cm [RR: 3.6 (3.3,3.9)], and family history [RR: 2.3 (2.2,2.5)] were also significantly associated with diabetes. For prediabetes, the main risk factors were age 50 to 65 years [RR: 2.6 (2.4,2.8)], BMI 30 kg/m^2^ or higher [RR: 1.9 (1.8,2.0)], hypertension and WC of 90 cm or higher [RR: 1.7 (1.6,1.8)]. The adjusted relative risks for all variables were higher in females than males, with the exception of family history for both conditions and waist circumference for prediabetes.

**Conclusions:**

Prediabetes and diabetes are prevalent in southwestern Iran. The major determinants are older age, obesity, and the presence of hypertension. Further interventions are required to escalate diabetes prevention and diagnosis in high-risk areas across Iran.

**Supplementary Information:**

The online version contains supplementary material available at 10.1186/s12902-021-00790-x.

## Background

The Middle East and North Africa (MENA) region holds the highest prevalence of diabetes mellitus (DM) worldwide, and is estimated to have the most significant increase in diabetes prevalence in the coming years [1]. However, there are limited population-based studies on the epidemiology and determinants of diabetes in MENA countries, including Iran [2].

By 2035, the number of adults with diabetes will increase by 55% globally, and the greatest proportional increase is expected in low-income, followed by low to middle-income countries [3]. The MENA region is estimated to have an over 95% increase in the number of people with diabetes by 2035 [4]. Nearly half of all diabetes deaths in this region occur among people under 60 years old; these premature deaths may be due to the new emerging risk factors, delayed diagnoses, and weak health-care systems that are not competent to bear the growing burden [5]. In addition, the absence of focused preventive measures, as well as lack of sufficient health insurance coverage, are among the major reasons underlying the high diabetes burden in MENA [6]. Aging populations, together with rapid urbanization that has brought major behavioral and lifestyle changes in many countries in this region, have resulted in a dramatic increase in diabetes prevalence [7]. Identifying the main traditional and non-traditional diabetes risk factors is crucial for developing preventive strategies and early diagnosis in such countries [2].

In Iran, the number of people with diabetes has been projected to increase by 91%, and its economic burden will increase markedly by 2035 [8, 9]. Despite the availability of medications and insulin coverage in Iran, utilization of antidiabetic drugs and control of blood glucose remains subpar [10–12]. According to previous national reports, only 13% of diabetes patients in Iran have controlled hyperglycemia [12]. Another Iranian study among patients living in urban areas reported that over one-third have hemoglobin A1c (HbA1c) levels higher than 9% [13]. In line with global initiatives to reduce the diabetes burden, Iran has made great prevention efforts in recent decades [14–16]. However, the rising trend of diabetes is outpacing such efforts and current role of diabetes risk factors remains poorly identified specially in remote areas across Iran [2, 12]. The present study aimed to investigate the prevalence and determinants of prediabetes and diabetes in a large population from southwestern Iran.

## Methods

### Study design

The Khuzestan comprehensive health study (KCHS) was conducted as a large cross-sectional study between October 2016 and November 2018 to assess the health conditions of 30,500 Iranian adults. The study site was Khuzestan province, located in southwestern Iran, which covers an area of more than 63,000 km^2^ and is home to a diverse population of nearly five million, including Fars, Arab, Bakhtiari, Kurd, and Lur ethnic groups. Written informed consent was obtained from all participants. All procedures performed in the study were in accordance with the ethical guidelines of the Declaration of Helsinki and the ethics committee of the National Institute for Medical Research Development (NIMAD) has approved the study protocol. The KCHS methodology has been detailed elsewhere [17].

### Study population

The study population includes all men and women aged 20 to 65 years, residing in urban and rural areas of Khuzestan province. The sample size required to estimate the prevalence of any health conditions in the province with 1% margin of error was calculated, and a 50% baseline prevalence to maximize the required sample size was assumed. Therefore, a sample size of 30,000 was chosen, which will provide a power greater than 90%. To obtain a representative sample, the population of all 27 counties in the province were stratified and a random sample of primary care centers -called health houses- in each county were chosen, which was proportional to total province population living in that county. Ultimately, 1079 random health houses were chosen and 30 individuals were randomly selected from the population covered by each center. Inclusion criteria were having been resided in Khuzestan for a minimum of 1 year prior to the study enrolment. Exclusion criteria were unwillingness to participate and any physical or psychological disability that hinder the participation.

### Study procedures

A multistage random sampling method was used to select participants. Trained staff went door to door to explain the study goals and invite the participants to the pre-defined study sites on the next day. The study sites include health centers, health houses, schools, and mosques, located in the close proximity of participants’ residential address. All participants provided written informed consent upon registration.

Fifteen mL of blood were obtained from each individual, divided into one clot tube and one EDTA tube. Blood was transferred to the reference lab within three hours of sampling, stored in Cool boxes at 4 °C. Blood samples were centrifuged at 3000 rpm for 10 min to separate serum; thereafter, serum level of fasting blood glucose (FBG) was measured by BT 1500 AutoAnalyzer, using commercial kits (Pars Azmoon, Iran). In case of any abnormal laboratory results, participants were referred to family physicians for further evaluation.

To reduce the measurement error, anthropometric data were gathered after blood sample collection, in a fasting state for 8 to 12 h. Height and weight were measured by the Seca 206 body meter measuring tape with Wall Stop and the Seca 762 mechanical flat scale, respectively, and the Seca 203 circumference measuring tape was used to measure waist circumference (WC). Blood pressure was measured using aneroid sphygmomanometers (Riester, Germany), twice from each arm in the sitting position. All measurements were in accordance with the US National Institutes of Health protocols. Data on demographics, socioeconomic status, and lifestyle habits were collected afterward by staff-administered questionnaires.

### Definition of variables and study outcomes

Diabetes was defined as having an FBG level of 126 mg/dl or higher, and/or taking antidiabetic medications, and/or self-declared diabetes. Prediabetes was defined as the FBG level of 100 or higher, but equal to or less than 125 mg/dl. General obesity was determined based on Body mass index (BMI), which is calculated by dividing weight (kg) by the square of the height (m) and categorized according to the World Health Organization (WHO) cut-off points [18]. BMI scores less than 25 kg/m^2^, between 25 and 29.9 kg/m^2^, and 30 kg/m^2^ or higher were defined as normal weight, overweight, and obese, respectively. Abdominal obesity was characterized by a WC of 90 cm or higher for both men and women [19]. Following major guidelines, hypertension (HTN) was defined as systolic blood pressure (SBP) equal to or higher than 140 mmHg, and/or diastolic blood pressure (DBP) equal to or higher than 90 mmHg, and/or taking antihypertensive medications [20]. Wealth score index was calculated using multiple correspondence analysis (MCA) with the following variables: house area, freezer, washing machine, dishwasher, laptop, internet, car type, TV type, indoor bathroom, vacuum cleaner, mobile use, and foreign travels [21].

### Statistical analysis

Data are described by the mean and standard deviation (SD) or frequency and percentage, where appropriate. A multinomial logistic regression model was used to assess the impact of the covariates on the risk of prediabetes or diabetes, which led to relative risks (RR) as the measure of association. In this model, individuals with prediabetes and diabetes were compared with normal participants. Each of the covariates sex, age, BMI, WC, HTN, family history of diabetes, and education have been put in the model, once alone and once adjusted for cigarette smoking (never/ever), hookah smoking (never/ever), opium use (never/ever), alcohol use (never/ever), marital status (single/married), residence (urban/rural), ethnicity (Arab/Bakhtiari/Fars/Turk/Kurd/Lur), and wealth index (four quartiles). These eight variables were selected as the adjustments since the preliminary assessments showed that the prevalence of prediabetes and diabetes did not differentiate between these variables’ strata. Two separate regression models were created to identify factors associated with prediabetes and diabetes in men and women. The analyses were performed using the statistical software Stata (version 12), and the chosen significance level was 0.05.

## Results

Overall, 30,498 individuals were included in the analysis, of whom 19,600 (64.3%) were women. The mean age of participants was 41.6 ± 11.9 years. The mean BMI and WC were 27.6 ± 5.3 kg/m^2^ and 92.5 ± 13.4 cm, respectively. Overall, the majority were married (83.0%), urban residents (73.1%), of Arab ethnicity (49.0%), and had primary education (44.2%). Eighty-nine percent were never-smokers, and cigarette smoking was reported among 28.0 and 1.3% of men and women, respectively. History of hookah and opium use was reported among 5.2 and 2.8%, respectively. Family history of diabetes was reported in 34.2% of all participants, and 19.7% were found to have HTN.

Among all participants, 9395 (30.8%) were identified to have prediabetes, and 4673 (15.3%) had diabetes. Diabetes had been self-reported among 2694 (57.7%) individuals, and 1941 (41.5%) had undiagnosed diabetes. The highest prevalence of diabetes was observed among people with HTN (33.5%), those who aged 50–65 years (32.3%), illiterates (24.7%), and those with a family history of diabetes (22.6%). For prediabetes, the highest prevalence was observed in people with BMI 30 kg/m^2^ or higher (34.6%), those with WC equal or more than 90 cm (33.2%), and the age range of 50–65 (32.9%) or 35–49.9 years (32.7%). Prevalence of diabetes was similar in both sexes (15.3%), while prediabetes was slightly higher in men (32.0 vs. 30.2%). Both conditions were more prevalent among urban residents and marrieds. People who belonged to the highest wealth index category and those of the Arab ethnicity had the highest prevalence for both diabetes (16.3 and 16.8%) and prediabetes (32.2 and 33.3%), respectively (Table 1). The standardized prevalence that was calculated according to the demographics, including sex, age, education, and BMI strata, did not change compared to the unadjusted prevalence of both conditions.

**Table 1:** Characteristics of the Khuzestan Comprehensive Health Study (KCHS) participants

Variable		Normal	Prediabetes	Diabetes
Number		16,430	9395	4673
Sex	Male	5750 (52.7%)	3483 (32.0%)	1665 (15.3%)
	Female	10,680 (54.5%)	5912 (30.2%)	3008 (15.3%)
Age (years)	20–34.9	7154 (69.6%)	2766 (26.9%)	353 (3.5%)
	35–49.9	6297 (53.9%)	3811 (32.7%)	1568 (13.4%)
	50–65	2950 (34.8%)	2797 (32.9%)	2745 (32.3%)
BMI (Kg/m^2^)	< 25	6343 (64.5%)	2593 (26.4%)	893 (9.1%)
	25–29.9	5991 (52.4%)	3616 (31.6%)	1833 (16.0%)
	≥30	4024 (44.3%)	3149 (34.6%)	1915 (21.1%)
Waist circumference (cm)	< 90	8052 (64.9%)	3404 (27.4%)	949 (7.7%)
	≥90	8306 (46.3%)	5955 (33.2%)	3692 (20.5%)
Hypertension	No	14,333 (58.5%)	7501 (30.6%)	2659 (10.9%)
	Yes	2089 (34.9%)	1891 (31.6%)	2013 (33.5%)
Diabetes family history	No	11,531 (57.6%)	6180 (30.9%)	2314 (11.5%)
	Yes	4867 (46.7%)	3201 (30.7%)	2350 (22.6%)
Education	Illiterate	2996 (45.3%)	1981 (30.0%)	1629 (24.7%)
	Elementary	7094 (52.6%)	4300 (31.9%)	2086 (15.5%)
	Secondary	4696 (59.9%)	2340 (29.8%)	808 (10.3%)
	Higher education	1642 (64.1%)	772 (30.1%)	149 (5.8%)
Cigarette smoking	Never	14,560 (53.8%)	8401 (31.1%)	4085 (15.1%)
	Ever	1784 (54.1%)	950 (28.8%)	564 (17.1%)
Hookah smoking	Never	15,435 (53.7%)	8853 (30.8%)	4461 (15.5%)
	Ever	909 (57.1%)	489 (30.7%)	195 (12.2%)
Opium use	Never	15,871 (53.8%)	9119 (30.9%)	4515 (15.3%)
	Ever	489 (57.5%)	229 (26.9%)	133 (15.6%)
Alcohol use	Never	15,936 (53.7%)	9135 (30.8%)	4589 (15.5%)
	Ever	406 (62.7%)	186 (28.7%)	56 (8.6%)
Marital status	Single	3050 (58.9%)	1559 (30.1%)	569 (11.0%)
	Married	13,380 (52.8%)	7836 (31.0%)	4104 (16.2%)
Ethnicity	Arab	7454 (49.9%)	4971 (33.3%)	2501 (16.8%)
	Bakhtiari	4236 (62.9%)	1668 (24.8%)	831 (12.3%)
	Fars	2984 (53.1%)	1726 (30.7%)	911 (16.2%)
	Turk/Kurd/Lur	1720 (54.6%)	1010 (32.0%)	422 (13.4%)
Residence	Urban	11,605 (52.1%)	7001 (31.4%)	3678 (16.5%)
	Rural	4824 (58.7%)	2394 (29.2%)	995 (12.1%)
Wealth index	Q_1_	4239 (55.5%)	2271 (29.7%)	1131 (14.8%)
	Q_2_	4018 (53.4%)	2340 (31.1%)	1161 (15.5%)
	Q_3_	4435 (54.8%)	2457 (30.4%)	1197 (14.8%)
	Q_4_	3641 (51.5%)	2274 (32.2%)	1152 (16.3%)

Results on multivariate analysis of the factors associated with diabetes and prediabetes risk are demonstrated in Table 2. For diabetes, the most significant risk factors include older age [RR (95% CI); 20.54 (18.13, 23.28)], HTN [RR (95%CI); 5.09 (4.72, 5.49)], and a WC of 90 cm or higher [RR (95% CI); 3.62 (3.34, 3.92)]. Moreover, compared to the participants with a BMI less than 25 kg/m^2^, those who had BMI 25–29.9 kg/m^2^ [RR (95% CI); 2.11 (1.93, 2.31)] or BMI equal to or higher than 30 kg/m^2^ [RR (95% CI); 3.24 (2.96, 3.56)] were more likely to be affected by diabetes. Having a history of diabetes in first-degree family member was associated with a two-fold higher likelihood of having diabetes [RR (95% CI); 2.30 (2.15, 2.46)], while higher education was associated with a lower risk [RR (95% CI); 0.10 (0.09, 0.13)].

Regarding prediabetes, the most significant risk factors were older age [RR (95% CI); 2.61 (2.43, 2.81)], BMI equal to 30 kg/m^2^ or higher [RR (95% CI); 1.88 (1.76, 2.01)], HTN [RR (95%CI); 1.71 (1.60,1.83)], and a WC of 90 cm or more [RR (95% CI); 1.66 (1.58, 1.76)]. Similar to diabetes, the highest educational degree was associated with a lower risk of prediabetes [RR (95% CI); 0.61 (0.54, 0.68)]. Furthermore, female sex was slightly protective against prediabetes [(RR (95% CI); 0.87 (0.82, 0.92)] (Table 2).

**Table 2:** Univariate and multivariate analysis of risk factors associated with diabetes and prediabetes in the Khuzestan study

Variable		Prediabetes		Diabetes	
		Unadjusted. RR (95% CI)	Adjusted. RR^*^ (95% CI)	Unadjusted. RR (95% CI)	Adjusted. RR^*^ (95% CI)
Sex	Male				
	Female	0.91 (0.87, 0.96)	0.87 (0.82, 0.92)	0.97 (0.91, 1.04)	0.98 (0.91, 1.06)
Age (years)	20–34.9				
	35–49.9	1.57 (1.47, 1.66)	1.63 (1.53, 1.73)	5.05 (4.47, 5.69)	5.25 (4.63, 5.95)
	50–65	2.45 (2.29, 2.62)	2.61 (2.43, 2.81)	18.86 (16.75, 21.24)	20.54 (18.13, 23.28)
BMI (Kg/m^2^)	< 25				
	25–29.9	1.48 (1.39, 1.57)	1.47 (1.38, 1.56)	2.17 (1.99, 2.37)	2.11 (1.93, 2.31)
	≥30	1.91 (1.79, 2.04)	1.88 (1.76, 2.01)	3.38 (3.09, 3.69)	3.24 (2.96, 3.56)
Waist circumference (cm)	< 90				
	≥90	1.70 (1.61, 1.79)	1.66 (1.58, 1.76)	3.77 (3.49, 4.08)	3.62 (3.34, 3.92)
Hypertension	No				
	Yes	1.73 (1.62, 1.85)	1.71 (1.60, 1.83)	5.19 (4.82, 5.59)	5.09 (4.72, 5.49)
Diabetes family history	No				
	Yes	1.23 (1.16, 1.30)	1.17 (1.11, 1.24)	2.41 (2.25, 2.57)	2.30 (2.15, 2.46)
Education	Illiterate				
	Elementary	0.92 (0.86, 0.98)	0.86 (0.80, 0.92)	0.54 (0.50, 0.58)	0.43 (0.40, 0.47)
	Secondary	0.75 (0.70, 0.81)	0.67 (0.62, 0.73)	0.32 (0.29, 0.35)	0.21 (0.19, 0.24)
	Higher education	0.71 (0.64, 0.79)	0.61 (0.54, 0.68)	0.17 (0.14, 0.20)	0.10 (0.09, 0.13)

^*^Data are adjusted for cigarette smoking (never vs. ever), hookah smoking (never vs. ever), opium use (never vs. ever), alcohol use (never vs. ever), marital status (single vs. married), residence (urban vs. rural), ethnicity (Arab vs. Bakhtiari vs. Fars vs. Turk/Kurd/Lur), and wealth index (four quartiles)

^*^RR Relative risk

Results on multivariate analysis of the investigated factors stratified by gender are presented in Tables 3 and 4. We observed a statistically significant difference for the association of diabetes and age among genders; the relative risk was more than two-fold higher for women with 50–65 years of age, compared to men in the same age range [RR: 25.02 vs. 11.56].

Among women, HTN had a higher relative risk for diabetes [RR: 6.46 vs. 3.00] and prediabetes [RR: 1.91 vs. 1.37] compared to men. Similarly, abdominal obesity in women was more significantly associated with diabetes [RR: 3.90 vs. 2.85]. Women with abdominal and general obesity had a similar likelihood of having diabetes [RR: 3.90 vs. 3.93], while among men, abdominal obesity had a higher relative risk for diabetes [RR: 2.85 vs. 2.47]. The relative risk for diabetes family history was slightly higher among men [RR: 2.49 vs. 2.25]. Regarding socioeconomic status, women with higher education had a lower relative risk for diabetes compared to men [RR: 0.03 vs. 0.27].

For prediabetes, the most significant risk factors were older age and general obesity between both men [RR: 1.82 and 1.72] and women [RR: 3.01 and 2.04], respectively, and older women had significantly higher relative risk than men of the same age. No significant difference was observed for the association of abdominal obesity and prediabetes among both genders (Tables 3 and4).

**Table 3:** Univariate and multivariate analysis of risk factors associated with diabetes and prediabetes among women in the Khuzestan study

Variables		Prediabetes		Diabetes	
		Unadjusted. RR (95% CI)	Adjusted. RR^*^ (95% CI)	Unadjusted. RR (95% CI)	Adjusted. RR^*^ (95% CI)
Age (years)	20–34.9				
	35–49.9	1.64 (1.52, 1.76)	1.69 (1.56, 1.82)	5.62 (4.83, 6.54)	5.83 (4.99, 6.81)
	50–65	2.85 (2.61, 3.10)	3.01 (2.75, 3.29)	23.2 (19.94, 26.99)	25.02 (21.42, 29.22)
BMI (Kg/m^2^)	< 25				
	25–29.9	1.44 (1.32, 1.56)	1.47 (1.36, 1.60)	2.43 (2.16, 2.73)	2.47 (2.18, 2.79)
	≥30	1.99 (1.84, 2.16)	2.04 (1.88, 2.22)	3.86 (3.44, 4.34)	3.93 (3.48, 4.43)
Waist circumference (cm)	< 90				
	≥90	1.62 (1.52, 1.73)	1.63 (1.53, 1.74)	3.87 (3.51, 4.25)	3.90 (3.54, 4.31)
Hypertension	No				
	Yes	1.91 (1.75, 2.08)	1.91 (1.74, 2.08)	6.54 (5.95, 7.18)	6.46 (5.87, 7.10)
Diabetes family history	No				
	Yes	1.22 (1.14, 1.30)	1.17 (1.09, 1.25)	2.31 (2.12, 2.51)	2.25 (2.07, 2.44)
Education	Illiterate				
	Elementary	0.87 (0.80, 0.94)	0.82 (0.75, 0.89)	0.49 (0.45, 0.54)	0.41 (0.37, 0.45)
	Secondary	0.66 (0.60, 0.73)	0.59 (0.53, 0.66)	0.23 (0.21, 0.27)	0.16 (0.14, 0.18)
	Higher education	0.58 (0.51, 0.66)	0.49 (0.43, 0.57)	0.05 (0.03, 0.07)	0.03 (0.02, 0.05)

^*^Data are adjusted for cigarette smoking (never vs. ever), hookah smoking (never vs. ever), opium use (never vs. ever), alcohol use (never vs. ever), marital status (single vs. married), residence (urban vs. rural), ethnicity (Arab vs. Bakhtiari vs. Fars vs. Turk/Kurd/Lur), and wealth index (four quartiles)

^*^RR Relative risk

**Table 4:** Univariate and multivariate analysis of risk factors associated with diabetes and prediabetes among men in the Khuzestan study

Variables		Prediabetes		Diabetes	
		Unadjusted. RR (95% CI)	Adjusted. RR^*^ (95% CI)	Unadjusted. RR (95% CI)	Adjusted. RR^*^ (95% CI)
Age (years)	20–34.9				
	35–49.9	1.44 (1.30, 1.59)	1.38 (1.23, 1.55)	4.13 (3.38, 5.03)	3.56 (2.85, 4.46)
	50–65	1.92 (1.72, 2.14)	1.82 (1.61, 2.07)	13.42 (11.08, 16.27)	11.56 (9.24, 14.47)
BMI (Kg/m^2^)	< 25				
	25–29.9	1.57 (1.42, 1.72)	1.46 (1.32, 1.61)	1.94 (1.71, 2.22)	1.66 (1.45, 1.90)
	≥30	1.88 (1.68, 2.12)	1.72 (1.52, 1.94)	3.02 (2.60, 3.50)	2.47 (2.11, 2.88)
Waist circumference (cm)	< 90				
	≥90	1.83 (1.68, 2.00)	1.66 (1.51, 1.82)	3.61 (3.16, 4.12)	2.85 (2.48, 3.27)
Hypertension	No				
	Yes	1.47 (1.32, 1.64)	1.37 (1.22, 1.53)	3.48 (3.08, 3.93)	3.00 (2.65, 3.41)
Diabetes family history	No				
	Yes	1.28 (1.16, 1.40)	1.23 (1.12, 1.36)	2.65 (2.37, 2.97)	2.49 (2.21, 2.80)
Education	Illiterate				
	Elementary	1.05 (0.92, 1.21)	0.97 (0.84, 1.12)	0.69 (0.59, 0.81)	0.55 (0.47, 0.65)
	Secondary	0.94 (0.82, 1.08)	0.85 (0.73, 1.00)	0.50 (0.42, 0.59)	0.37 (0.31, 0.45)
	Higher education	0.97 (0.81, 1.15)	0.84 (0.69, 1.02)	0.40 (0.32, 0.51)	0.27 (0.21, 0.36)

^*^Data are adjusted for cigarette smoking (never vs. ever), hookah smoking (never vs. ever), opium use (never vs. ever), alcohol use (never vs. ever), marital status (single vs. married), residence (urban vs. rural), ethnicity (Arab vs. Bakhtiari vs. Fars vs. Turk/Kurd/Lur), and wealth index (four quartiles)

^*^RR Relative risk

We further evaluated the characteristics of the normal weight and underweight participants affected by diabetes (Table 5). Overall, 893 individuals with BMI less than 25 kg/m^2^ were identified to have diabetes; the mean age was 50.4 ± 10.8 years and 50.2% were female. The mean WC in this group was 86.1 ± 8.8 cm, mostly had a WC less than 90 cm (62.8%). The majority aged 50–65 years (61.1%), were married (85.7%) and were urban residents (71.8%). Most of these individuals had no history of HTN (64.3%) and smoking (82.1%), while nearly half had a family history of diabetes (48.1%) and one-third belonged to the lowest wealth index category (29.9%). (Table 5).

**Table 5:** Characteristics of the Khuzestan study participants with diabetes who had Body Mass Index (BMI) less than 25 kg/m2

Variable		Number (%)
People with diabetes and BMI < 25 kg/m2		893
Sex	Male	445 (49.8%)
	Female	448 (50.2%)
Age (years)	20–34.9	99 (11.1%)
	35–49.9	248 (27.8%)
	50–65	545 (61.1%)
Waist circumference (cm)	< 90	561 (62.8%)
	≥90	332 (37.2%)
Hypertension	No	574 (64.3%)
	Yes	319 (35.7%)
Diabetes family history	No	463 (51.9%)
	Yes	429 (48.1%)
Education	Illiterate	309 (34.6%)
	Elementary	396 (44.4%)
	Secondary	161 (18.1%)
	Higher education	26 (2.9%)
Cigarette smoking	Never	725 (82.1%)
	Ever	158 (17.9%)
Hookah smoking	Never	843 (94.7%)
	Ever	47 (5.3%)
Opium use	Never	842 (94.7%)
	Ever	47 (5.3%)
Alcohol use	Never	869 (97.6%)
	Ever	2 (2.4%)
Marital Status	Single	127 (14.3%)
	Married	760 (85.7%)
Residence	Urban	637 (71.8%)
	Rural	250 (28.2%)
Wealth index	Q_1_	265 (29.9%)
	Q_2_	228 (25.7%)
	Q_3_	223 (25.2%)
	Q_4_	170 (19.2%)

## Discussion

This study investigates the prevalence and determinants of prediabetes and diabetes in a population-based sample of Iranian adults residing in southwestern Iran. Prediabetes with 30.8% and diabetes with 15.3% were more prevalent in this area than in most other parts of the country. In line with some previous Iranian studies, older age, hypertension, and obesity were the most significant risk factors for diabetes and prediabetes [22] [23]. Also, family history of diabetes was associated with a two-fold higher risk of diabetes. These findings indicate that urgent control measures are needed to impede the high burden of diabetes in this region of Iran.

The prevalence of diabetes in this study was identical to a survey conducted by Yazdanpanah et al. in 2015, which is the only available report on diabetes from southwestern Iran (15.3% vs. 15.1%) [24], and was comparable to two recent studies from central (16.1%) [25] and Northern Iran (17.2%) [9]. However, this prevalence was nearly two-fold higher compared to the national estimations [26] and other parts of the country, including western [27], southeastern [28], and southern areas [29]. For prediabetes, two population-based studies in central and southeastern Iran have reported a lower prevalence (26 and 19%); however, in both studies, individuals under the age of 35 years were also recruited [25]. These comparisons indicate that diabetes is exceptionally prevalent in the southwestern region of Iran, and urgent interventions should be taken to scale-up focused preventive and diagnostic programs [15]. Also, people of Arab ethnicity had the highest prevalence of both conditions. Given the subpar control of hyperglycemia in Iran, therapeutic interventions should be particularly expanded into the most high-risk populations and ethnic groups [12].

Globally, elderly people are at substantial risk for diabetes [30]. In this study, one-third of individuals aged 50–65 years had diabetes, which is similar to the results of an elderly-based survey conducted among people above 60 years in southern Iran; however, their reported prevalence for prediabetes was two-fold higher than our estimation for elderlies (67% vs. 33%) [31]. Although older age was also the most significant risk factor for prediabetes, its relative risk was much lower compared to diabetes (2.4 vs. 18.5). Besides, older women had more than two-fold higher the relative risk of diabetes than men across the age range (25.0 vs. 11.6), which can be explained by the large sex differences in obesity rates, especially after the age of 45 years and in countries with greater gender inequality [32, 33].

The second significant diabetes risk factor was HTN, having a five-fold higher relative risk in people with HTN than those without such condition, and a two-fold higher relative risk for women compared to men. This observation is in line with previous global reports [34]. Regarding obesity, some studies have emphasized the benefits of WC compared to BMI in predicting diabetes in the Iranian population [1, 35]. In our study, WC showed a stronger association than BMI for diabetes (3.6 vs. 3.2), but not for prediabetes (1.7 vs. 1.9). Existing evidence has shown that men are diagnosed with diabetes at lower BMI than women of the same age group [36]. Accordingly, we observed no difference between WC and BMI among women for the risk of diabetes; however, BMI had a lower relative risk in men.

A study from Northern Iran showed that having one or more than one family member with diabetes increases the odds of this disease to 2.3 and 4.2, respectively [9]. Another cohort from the center of Iran showed that people with a first-degree family history were 3.3 times more likely to have the disease [37]. Similarly, family history in first-degree relatives, having a relative risk equal to 2.4, was among the significant risk factors for diabetes in our study, with a slightly higher relative risk in men. Also, from people with diabetes and normal BMI, approximately half had a family history of diabetes, and the majority were from the lower socioeconomic categories. In a study conducted on over 9000 Iranian adults, Mirzaei et al. have found a negative interaction between family history of diabetes and other risk factors only for BMI [38]. Their results suggested that despite considering family history as an independent risk factor, as long as it is used as a tool to raise awareness and promote lifestyle changes, it would reduce the risk of developing diabetes in people with other risk factors such as obesity.

Evidence on the modifying effect of sex in the development of diabetes is controversial. Several Iranian studies have reported a higher prevalence among females [1, 39, 40], and some others have not found any significant associations for sex [41]. Similar to the study of Yazdanpanah et al. from southwestern Iran, we observed a similar prevalence of diabetes in both sexes. Moreover, in our study, the female sex was slightly protective against prediabetes. According to a recent study, obesity is the most significant risk factor for sex inequalities in type 2 diabetes in Iranians and can partly explain the observed differences in the prevalence of diabetes between sexes [42].

Many studies have confirmed the role of socioeconomic factors in developing diabetes [43]. We observed higher diabetes and prediabetes prevalence among urban residents and illiterates. In fact, any level of education, mostly higher education, appears to be a protective factor for both diabetes and prediabetes. We also observed that higher education is more protective for diabetes among women, compared to men. In total, 8.4% of participants have had higher education, while only 2.9% of participants with diabetes and BMI < 25 reported higher education. This observation suggests that lower education has bidirectional effects, both on its association with BMI < 25, and with higher prevalence of diabetes. These findings were in line with other studies on socioeconomic inequalities and diabetes in Iran [25, 44].

Although many studies indicate that smoking increases the risk of diabetes, some evidence supports the reverse association [45]. In our study, diabetes was more prevalent in ever-smokers, while for prediabetes a higher prevalence was observed among never-smokers. Also, studies have suggested that opium may temporarily decrease blood glucose [46]. Among people who had a history of opium use in the present study, the prevalence of prediabetes was slightly higher compared to those who had never used; however, for diabetes the prevalence was similar among both groups. Therefore, the association of cigarette and opium use with diabetes in the Iranian population needs further investigation.

### Limitations

In this study, people above the age of 65 years were not included, which may have been led to a lower estimation of diabetes prevalence. Besides, using only one method for diagnosis and the unavailability of oral glucose tolerance test (OGTT) and HbA1c testing might be other causes of underestimating the prevalence of diabetes in this region of Iran. However, in many epidemiological studies in the region, there is significant discordance between OGTT and FBG-based prediabetes and diabetes diagnoses, and the prevalence of newly diagnosed diabetes is about 15 to 20% higher according to the FBG criteria [47]. The cross-sectional design and higher participation rate among women and housewives were among the other limitations of this study. Also, we were unable to exclude Type 1 diabetes from our analysis.

## Conclusions

Prediabetes and diabetes are prevalent in southwestern Iran, and their prevalence is higher than in most parts of the country. The major determinants are older age, the presence of hypertension, and obesity. Further studies are needed to better understand the epidemiology of diabetes across Iran, and urgent interventions should be applied to escalate diabetes prevention, diagnosis, and treatment among high-risk populations residing in Iran.

## Supplementary Information

**Additional file 1: Supplementary table.** Distribution of people who were diagnosed with diabetes in the Khuzestan Comprehensive Health Study (KCHS), *n* = 4673

## Data Availability

The datasets used and/or analysed during the current study are available from the corresponding author on reasonable request.

## Abbreviations

BMI: Body mass index
DBP: Diastolic blood pressure
DM: Diabetes mellitus
FBG: Fasting blood glucose
HbA1c: Hemoglobin A1c
HTN: Hypertension
KCHS: Khuzestan Comprehensive Health Study
MCA: Multiple correspondence analysis
MENA: Middle East and North Africa
NIMAD: National Institute for Medical Research Development
OGTT: Oral glucose tolerance test
RR: Relative risk
SBP: Systolic blood pressure
SD: Standard deviation
WC: Waist circumference
WHO: World Health Organization
