# Supplementary material for: Prevalence and determinants of diabetes and prediabetes in southwestern Iran: the Khuzestan comprehensive health study (KCHS)
Source: BMC Endocr Disord. 2021 Jun 29;21:135. doi: 10.1186/s12902-021-00790-x (PMC8243419; doi:10.1186/s12902-021-00790-x)
Supplement: Supplementary file 1 — Additional file 1: Supplementary table. Distribution of people who were diagnosed with diabetes in the Khuzestan Comprehensive Health Study (KCHS), n = 4673 [file 12902_2021_790_MOESM1_ESM.docx]

| Taking antidiabetic medication | Self-declared diabetes | FBG≥126 | Number (%) |
| --- | --- | --- | --- |
| Yes | Yes | Yes | 450 (9.6 %) |
| Yes | Yes | No | 125 (2.7 %) |
| Yes | No | Yes | 11 (0.2 %) |
| Yes | No | No | 27 (0.6 %) |
| No | Yes | Yes | 1612 (34.5 %) |
| No | Yes | No | 507 (10.9 %) |
| No | No | Yes | 1941 (41.5 %) |

Supplementary table. Distribution of people who were diagnosed with diabetes in the Khuzestan Comprehensive Health Study (KCHS), n=4,673
